# Complex Fractures of the Tibia and Femur Treated with Static Interlocking Intramedullary Nail

**Published:** 2011-03-01

**Authors:** S Solooki, S A R Mesbahi

**Affiliations:** 1 Department of Orthopaedic Surgery, Shiraz University of Medical Sciences, Shiraz, Iran; 2 Orthopaedic Surgeon, Bandarabbas, Iran

**Keywords:** Complex fractures, Dynamization, Intramedulary nailing

## Abstract

**Background:**

Reamed interlocking intramedullary nailing is considered the gold standard treatment for complex fractures of the femoral and tibial shaft. There has been some controversies about dynamization of statically locked nails, and some authors recommended routine dynamization for promotion of healing. This study aims to evaluate treatment of complex fractures in tibia and femur with static interlocking intramedullary nail method.

**Methods:**

In a retrospective study from January 2003 to April 2008, 173 patients with femoral and tibial shaft fracture that were treated with this method were enrolled. No rod was dynamized in our patients.

**Results:**

All patients with tibial fractures achieved union without any need for dynamization during 12-18 weeks (mean; 13.4 weeks). Four patients developed delayed union but all achieved union without any intervention. In femoral fracture, all but one patient achieved complete union during 10-30 weeks (mean: 18.3 weeks). One patient developed non-union who was treated by an exchange nailing and iliac bone graft method. No significant complication was observed in our patients.

**Conclusion:**

It is not necessary to routinely dynamize nails in tibial and femoral shaft fractures as all fractures united in acceptable alignment without any complication.

## Introduction

Open reamed interlocking intramedulary nailing is the preferred treatment option for complex femoral and tibia shaft fractures.[1][2][3][4][5].Some authors recommended routine dynamization of static interlocking nails to promote healing.[6][7]. Even complex femoral and tibia shaft fractures treated with static interlocking nailing without dynamization have been reported to have union rates as high as 98 to 100%.[4][8][9]. The results of previous works gave rise to the question if dynamization of static interlocking nailing of femoral and tibial fracture is always necessary?

The aim of this retrospective study was to help resolving this controversy and determine the union rate of complex femoral and tibia shaft fractures treated with static interlocking nailing without dynamization.

## Materials and Methods

From January of 2003 to April 2008, 61 patients (53 males and 8 females) with complex closed fracture of tibial shaft and 112 patients (101 males and 11 females) with complex closed fracture of femoral shaft were treated with static proximal and distal interlocking intramedullary nails. All patients developed fractures due to high energy trauma (a motor vehicle accident or falling from the height) and all underwent surgery for treatment of fracture soon after a systemic condition that was stabilized. Our tibial fracture patients were 17 to 70 years old (mean: 28.29) and our femoral fracture patients aged from 17 to 75 years old (mean: 26.57). In tibial fracture group, 33 patients had right sided fracture and 28 had a left sided one. In femoral fracture group, 59 patients had right sided fracture and 53 had a left sided one. All patients had comminuted fracture (Winquist type III or IV). In tibial fracture group, 37 patients had fracture in the middle third of tibial shaft; 9 patient had fracture in proximal third and 2 patients had fracture in distal third of tibial. Thirteen patients had segmental pattern of fracture.In femoral shaft fracture group, 66 patients had fracture in middle third; 16 patients in proximal third and 2 patients in distal third and 28 patients had segmental fracture in the femur. In femoral fracture group, 46 patients and in tibial fracture group, 25 patients had other associated injuries that required specific surgical or medical care (eg: internal bleeding; head trauma etc.). All patients underwent static interlocking intramedulary nailing as soon as general condition of patients was stabilized. In all patients, open reduction was performed under general anesthesia and the canals were reamed as large as possible (usually 13-14 mm in diameter) and an adequate size static interlocking nail was inserted (usually 12 or 13 mm in diameter). A proximal interlocking was performed with specific interlocking guides and a distal locking under an image intersifier. Post-operatively, the patients started partial weight bearing ambulation as early as possible (usually after 48 hours post-operation). An isometric quadriceps exercise and a range of motion in the knee, hip and ankle were encouraged. The patients were followed at the OPD clinic by the same surgeon at 3-6 week intervals until complete union was achieved. The clinical and roentgenographic signs of healing process were recorded.

All patients were followed for at least for 9 months (range: 9-36 months). Fracture union was defined as follows: Clinically there was no tenderness or pain and fracture had no motion and patients were walking without any walking aids. Roentgenographically, solid bridging callus with cortical density connected fracture fragments in at least 3 from 4 cortices. Delayed union was defined as an incomplete healing during a 6 months period after the fracture fixation.

## Results

All patients with tibial fracture achieved union without any need for dynamization or any surgical intervention during 12-28 weeks (mean: 13.4 weeks). Two patients with tibial fracture developed wound infection that improved with antibiotic treatment. Four patients in tibial fracture group developed delayed union but all of them achieved union completely without any surgical intervention up to 28 weeks. Alignment of tibial fracture was perfect in all patients without any shortening and rotation. In femoral fracture group, all but one patients achieved complete union during 10-30 weeks (mean: 18.3 weeks). There was no significant complication in femoral fracture group. Seven patients with femoral fracture developed delayed union that were completely united by 24-30 weeks after fracture fixation without any surgical intervention. One patient developed non-union that was treated with changing of the nail and iliac crest bone graft after 36 weeks of primary surgery and achieved union by 16 weeks after bone grafting. Alignment of femoral fracture was perfect without any significant shortening and rotation in our patients with femoral shaft fracture.

## Discussion

Intramedullary nailing has become the treatment of choice for fractures of the femoral and tibial shaft.[1][2][3][4][9]. In complex and comminuted fractures of femoral and tibial shaft, it is not possible to dynamize the intramedullary nailing because of concern about shortening and rotational instability.[4][10][11] So it is mandatory to use interlocking intramedullary nails in such fractures.

During recent years, many authors recommended dynamization for promotion of healing in statically locked intramedullary nails of femoral or tibial diaphyseal fractures.[5][6][9][12][13]. In review of articles, we found that some complications may occur after dynamization of a statically locked intramedullary nailing such as loss of length and rotational malalignments, [5] so we reviewed the results of treatment of femoral and tibial shaft fractures in 173 patients who underwent statically locked intramedullary nailing. In a retrospective study in Taiwan, 220 acute complex femoral shaft fractures were treated with static interlocking nails, 28 nails had been dynamized during treatment course and 5 patients from this dynamization group developed significant (more than 2 cm) shortening, [5] and they reported only a 58% union rate achieved after dynamization. In another study performed in India, no significant promotion of healing was observed in 26 patients out of 50 dynamization cases of statically locked IM nailing. [6]

In a retrospective study in Taiwan, the success rate of dynamization was only 54%.[14] In another retrospective study done in Italy, time to union was significantly shorter in the static group compared with dynamized group (103 days compared with 126 days).[7] According to these problems in dynamization (complications and no significant promotion of healing following dynamization), and according to our results that revealed a high union rate of complex femoral and tibial shaft fracture (about 99% union rate), it is better to treat complex femoral and tibial shaft fracture with static intramedullary nailing method without any dynamization and if non-union and delayed union occur, these patients can be treated with other options other than dynamization. Although, we did not any have control group (dynamization group) for comparison of time to union, it seems that time to union probably is not different significantly following dynamization.
